# Acute mesenteric ischemia: guidelines of the World Society of Emergency Surgery

**DOI:** 10.1186/s13017-017-0150-5

**Published:** 2017-08-07

**Authors:** Miklosh Bala, Jeffry Kashuk, Ernest E. Moore, Yoram Kluger, Walter Biffl, Carlos Augusto Gomes, Offir Ben-Ishay, Chen Rubinstein, Zsolt J. Balogh, Ian Civil, Federico Coccolini, Ari Leppaniemi, Andrew Peitzman, Luca Ansaloni, Michael Sugrue, Massimo Sartelli, Salomone Di Saverio, Gustavo P. Fraga, Fausto Catena

**Affiliations:** 1 Acute Care Surgery and Trauma Unit, General Surgery Department, Hadassah - Hebrew University Medical Center, Kiriat Hadassah, POB 12000, 91120 Jerusalem, Israel; 2 Assia Medical Group, Tel Aviv University Sackler School of Medicine, Tel Aviv, Israel; 3 Department of Surgery, Denver Health Medical Center, University of Colorado, Denver, USA; 4 Department of General Surgery, Rambam Health Care Campus, Haifa, Israel; 5 Department of Surgery, Queens Medical Center, Honolulu, Hi USA; 6 Faculdade de Ciências Médicas e da Saúde de Juiz de Fora (SUPREMA), Federal University of Juiz de Fora (UFJF), Juiz de Fora, MG Brazil; 7 Department of Vascular Surgery, Hadassah Hebrew University Medical Center, Jerusalem, Israel; 8 Department of Traumatology, John Hunter Hospital and University of Newcastle, Newcastle, NSW Australia; 9 Department of Surgery, Auckland City Hospital, Auckland, New Zealand; 10 General Surgery I, Papa Giovanni XXIII Hospital, Bergamo, Italy; 11 Abdominal Center, University Hospital Meilahti, Helsinki, Finland; 12 Department of Surgery, UPMC, University of Pittsburgh School of Medicine, Pittsburgh, PA USA; 13 Donegal Clinical Research Academy, Letterkenny University Hospital, Letterkenny, Ireland; 14 Department of Surgery, Macerata Hospital, Macerata, Italy; 15 Trauma Surgery Unit, Maggiore Hospital, Bologna, Italy; 16 Division of Trauma Surgery, Hospital de Clinica, School of Medical Sciences, University of Campinas, Campinas, Brazil; 17 Emergency Department, Maggiore University Hospital, Parma, Italy

**Keywords:** Mesenteric ischemia, Mesenteric arterial occlusion, Mesenteric angiography, Mesenteric artery stenting, Small bowel ischemia, Guidelines, Recommendations

## Abstract

Acute mesenteric ischemia (AMI) is typically defined as a group of diseases characterized by an interruption of the blood supply to varying portions of the small intestine, leading to ischemia and secondary inflammatory changes. If untreated, this process will eventuate in life threatening intestinal necrosis. The incidence is low, estimated at 0.09–0.2% of all acute surgical admissions. Therefore, although the entity is an uncommon cause of abdominal pain, diligence is always required because if untreated, mortality has consistently been reported in the range of 50%. Early diagnosis and timely surgical intervention are the cornerstones of modern treatment and are essential to reduce the high mortality associated with this entity. The advent of endovascular approaches in parallel with modern imaging techniques may provide new options. Thus, we believe that a current position paper from World Society of Emergency Surgery (WSES) is warranted, in order to put forth the most recent and practical recommendations for diagnosis and treatment of AMI. This review will address the concepts of AMI with the aim of focusing on specific areas where early diagnosis and management hold the strongest potential for improving outcomes in this disease process.

Some of the key points include the prompt use of CT angiography to establish the diagnosis, evaluation of the potential for revascularization to re-establish blood flow to ischemic bowel, resection of necrotic intestine, and use of damage control techniques when appropriate to allow for re-assessment of bowel viability prior to definitive anastomosis and abdominal closure.

## Background

Acute mesenteric ischemia (AMI) may be defined as a sudden interruption of the blood supply to a segment of the small intestine, leading to ischemia, cellular damage, intestinal necrosis, and eventually patient death if untreated [1]. AMI may be non-occlusive (NOMI) or occlusive, with the primary etiology further defined as mesenteric arterial embolism (50%), mesenteric arterial thrombosis (15–25%), or mesenteric venous thrombosis (5–15%) [2, 3]. The overall incidence is low (0.09 to 0.2% of all acute admissions to emergency departments), representing an uncommon cause of abdominal pain [4–6]. Prompt diagnostic and intervention are essential to reduce the high mortality rates (50 to 80%) [7–10].

There are currently no level 1 evidence to guide the evaluation and treatment of suspected AMI, and the published literature contains primarily institutional reviews, case series and personal recommendations with no clearly defined treatment guidelines.

Accordingly, this review aims to provide an update with recommendations based on the most currently accepted concepts in the management of AMI.

The current presentation evolved from the contributions of a group of experts in the field who submitted their evidence-based literature review of key points pertaining to diagnosis and management of AMI. Following preliminary preparation of these key points, a coordinated presentation was organized during the WSES World Congress, May 2017 in Campinas, Brazil. The final version has taken into account the presentations at the congress as well as pertinent group discussions and comments on the various presentations.

The grading of recommendations was evaluated (Table 1).

**Table 1:** Grading of recommendations

Grade of recommendation	Clarity of risk/benefit	Quality of supporting evidence	Implications
1A			
Strong recommendation, high-quality evidence	Benefits clearly outweigh risk and burdens, or vice versa	RCTs without important limitations or overwhelming evidence from observational studies	Strong recommendation, applies to most patients in most circumstances without reservation
1B			
Strong recommendation, moderate-quality evidence	Benefits clearly outweigh risk and burdens, or vice versa	RCTs with important limitations (inconsistent results, methodological flaws, indirect analyses, or imprecise conclusions) or exceptionally strong evidence from observational studies	Strong recommendation, applies to most patients in most circumstances without reservation
1C			
Strong recommendation, low-quality or very low-quality evidence	Benefits clearly outweigh risk and burdens, or vice versa	Observational studies or case series	Strong recommendation but subject to change when higher quality evidence becomes available
2A			
Weak recommendation, high-quality evidence	Benefits closely balanced with risks and burden	RCTs without important limitations or overwhelming evidence from observational studies	Weak recommendation, best action may differ depending on the patient, treatment circumstances, or social values
2B			
Weak recommendation, moderate-quality evidence	Benefits closely balanced with risks and burden	RCTs with important limitations (inconsistent results, methodological flaws, indirect or imprecise) or exceptionally strong evidence from observational studies	Weak recommendation, best action may differ depending on the patient, treatment circumstances, or social values
2C			
Weak recommendation, low-quality or very low-quality evidence	Uncertainty in the estimates of benefits, risks, and burden; benefits, risk, and burden may be closely balanced	Observational studies or case series	Very weak recommendation; alternative treatments may be equally reasonable and merit consideration

### Mesenteric vascular anatomy and physiology

The superior mesenteric artery (SMA) is the primary blood supply for the small bowel with some collateral flow from the celiac arterial system, via the superior and inferior pancreaticoduodenal arteries, as well as from the inferior mesenteric artery. Intestinal blood returns via the portal vein. The splanchnic circulation receives 15–35% of the cardiac output, depending on the feeding state, but oxygen extraction is relatively low, accounting for the oxygen delivery capacity of the portal vein to the liver. Thus, blood supply must be reduced by more than 50% before the small intestine becomes ischemic [11].

Furthermore, the intestines can autoregulate oxygen availability via enhanced oxygen extraction and perfusion due to vasodilation. Experimentally, it had been shown that mesenteric ischemia does not occur until the patient’s mean arterial pressure is <45 mmHg [12]. Consequently, the small intestine is able to compensate for a 75% reduction in mesenteric blood flow for up to 12 h [13].

### Pathophysiology and epidemiology

#### Acute mesenteric arterial embolism

Roughly, 50% of all cases of AMI are due to acute mesenteric embolism [2, 3]. Mesenteric emboli can originate from the left atrium, associated with cardiac dysrhythmias such as atrial fibrillation, left ventricle with global myocardial dysfunction associated with poor ejection fraction, or cardiac valves due to endocarditis. Occasionally emboli generated from an atherosclerotic aorta. Emboli typically lodge at points of normal anatomic narrowing, and the SMA is particularly vulnerable because of its relatively large diameter and low takeoff angle from the aorta. The majority of emboli lodge 3 to 10 cm distal to the origin of the SMA, thus classically sparing the proximal jejunum and colon. More than 20% of emboli to the SMA are associated with concurrent emboli to another arterial bed including the spleen, or kidney. Thus, findings of changes in these organs on CTA suggest a proximal embolic source [14].

#### Acute mesenteric arterial thrombosis

Thrombosis of the SMA (approximately 25% of cases) is usually associated with pre-existing chronic atherosclerotic disease leading to stenosis. Many of these patients have a history consistent with chronic mesenteric ischemia (CMI), including postprandial pain, weight loss, or “food fear”, and thus a systematic history is important when evaluating a patient suspected to have AMI. Thrombosis usually occurs at the origin of visceral arteries, moreover, an underlying plaque in the SMA usually progresses to a critical stenosis over years resulting in collateral beds. Accordingly, symptomatic SMA thrombosis most often accompanies celiac occlusion [15]. SMA thrombosis may also occur due to vasculitis, mesenteric dissection, or a mycotic aneurysm. Involvement of the ileocolic artery will result in necrosis of the proximal colon.

#### Pathophysiology of acute non-occlusive mesenteric ischemia

NOMI occurs in approximately 20% of cases and is usually a consequence of SMA vasoconstriction associated with low splanchnic blood flow [16]. The compromised SMA blood flow often involves the proximal colon as well due to involvement of the ileocolic artery. Patients with NOMI typically suffer from severe coexisting illness, commonly cardiac failure which may be precipitated by sepsis. Hypovolemia and the use of vasoconstrictive agents may precipitate NOMI.

#### Mesenteric venous thrombosis

Mesenteric venous thrombosis (MVT) accounts for less than 10% of cases of mesenteric infarction. Thrombosis is attributed to a combination of Virchow’s triad, i.e., stagnated blood flow, hypercoagulability, and vascular inflammation, but approximately 20% are idiopathic. Hypercoagulabilty may be due to inherited disease such as Factor V Leiden, prothrombin mutation, protein S deficiency, protein C deficiency, antithrombin deficiency, and antiphospholipid syndrome. Additionally, recent work suggests that fibrinolysis shutdown (resistance to tissue plasminogen activator (tPA)) is a significant risk factor for hypercoagulability [17]. Thrombophilia may also be acquired due to malignancies, hematologic disorders, and oral contraceptives [18].

The additional components altering blood flow include portal hypertension, pancreatitis, inflammatory bowel disease, sepsis, and trauma. In these situations, the consequences of bowel edema and increased vascular resistance secondary to venous thrombosis result in reduced arterial blood flow, leading to bowel ischemia.

## Severe abdominal pain out of proportion to physical examination findings should be assumed to be AMI until disproven. (Recommendation 1B)

The key to early diagnosis is a high level of clinical suspicion.

The clinical scenario of a patient complaining of excruciating abdominal pain with an unrevealing abdominal exam is classic for early AMI [19]. If the physical exam demonstrates signs of peritonitis, there is likely irreversible intestinal ischemia with bowel necrosis. In a study of AMI, 95% of patients presented with abdominal pain, 44% with nausea, 35% with vomiting, 35% with diarrhea, and 16% with blood per rectum [20]. Approximately one-third of patients present with the triad of abdominal pain, fever, and hemocult-positive stools. Other patients, particularly those with delayed diagnosis, may present in extremis with septic shock. Clinical signs of peritonitis may be subtle. Accordingly, one must have a high index of suspicion, because such findings almost always are predictive of intestinal infarction.

## Clinical scenario differentiates AMI as mesenteric arterial emboli, mesenteric arterial thrombosis, NOMI or mesenteric venous thrombosis. (Recommendation 1B)

### Phenotypes of AMI

A careful history is important because distinct clinical scenarios are associated with the pathophysiological form of AMI [21]. Patients with mesenteric arterial thrombosis often have a history of chronic postprandial abdominal pain, progressive weight loss, and previous revascularization procedures for mesenteric arterial occlusion. Patients with NOMI have pain that is generally more diffuse and episodic associated with poor cardiac performance. Patients with MVT present with a mixture of nausea, vomiting, diarrhea, and abdominal cramping. Gastrointestinal bleeding occurs in 10% [22].

Nearly 50% of patients presenting with embolic AMI have atrial fibrillation and approximately one-third of patients have a prior history of arterial embolus [20].

Risk factors for specific phenotypes of AMI presented in Table 2.

**Table 2:** Risk factors for specific phenotypes of AMI

	Pathogenesis of AMI			
	Acute mesenteric arterial embolism	Acute mesenteric arterial thrombosis	NOMI	Mesenteric venous thrombosis
Risk factors	Atrial fibrillation Recent MI cardiac thrombi Mitral valve disease Left ventricular aneurysm Endocarditis Previous embolic disease	Diffuse atherosclerotic disease Postprandial pain Weight loss	Cardiac failure Low flow states Multi-organ dysfunction Vasopressors	Portal hypertension History of VTE Oral contraceptives Estrogen use Thrombophilia pancreatitis

*AMI* acute mesenteric ischemia, *NOMI* non-occlusive mesenteric ischemia, *MI* myocardial infarction, *VTE* venous thromboembolism

## Conventional plain X-ray films have limited diagnostic value in evaluating AMI, although signs of intestinal perforation may be seen. (Recommendation 1B)

A radiograph is usually the initial test ordered in patients with acute abdominal pain but has a limited role in the diagnosis of mesenteric ischemia, especially in the early setting. A negative radiograph does not exclude mesenteric ischemia [23]. Plain radiography only becomes positive when bowel infarction has developed and intestinal perforation manifests as free intraperitoneal air.

## There are no laboratory studies that are sufficiently accurate to identify the presence or absence of ischemic or necrotic bowel, although elevated l-lactate, and D-dimer may assist. (Recommendation 1B)

Although laboratory results are not definitive, they may help to corroborate clinical suspicion. More than 90% of patients will have an abnormally elevated leukocyte count. The second most commonly encountered abnormal finding is metabolic acidosis with elevated lactate level, which occurred in 88% [24].

Patients may present with lactic acidosis due to dehydration and decreased oral intake. Thus, differentiation of early ischemia versus irreversible bowel injury based upon the lactate level alone is not reliable unless accompanied by other clinical evidence. Elevated serum lactate levels >2 mmol/l was associated in irreversible intestinal ischemia (Hazard Ratio: 4.1 (95% CI: 1.4–11.5; *p* < 0.01) in established diagnosis of AMI [25].

It should be emphsized that the presence of lactic acidosis in combination of abdominal pain when the patient may not otherwise appear clinically ill should lead to consideration for early CTA.

Based on the current literature, no accurate biomarkers have been identified to date [26, 27]. D-dimer has been reported to be an independent risk factor of intestinal ischemia [27], reflecting ongoing clot formation and endogenous degradation via fibrinolysis. No patient presenting with a normal D-dimer had intestinal ischemia and D-dimer >0.9 mg/L had a specificity, sensitivity, and accuracy of 82, 60, and 79%, respectively [28]. Thus, D-dimer may well be useful in the early assessment. Elevated amylase has been reported in roughly a half of patients with AMI [29]. Other biomarkers reported to assist in the diagnosis of AMI include intestinal fatty acid binding protein (I-FABP), serum alpha-glutathione S-transferase (alpha–GST), and cobalt-albumin binding assay (CABA) [30, 31]. These biomarkers may offer improved diagnostic accuracy of acute mesenteric ischemia, however, further research is required to specify its accuracy and values.

## Computed tomography angiography (CTA) should be performed as soon as possible for any patient with suspicion for AMI. (Recommendation 1A)

**Fig. 1:**
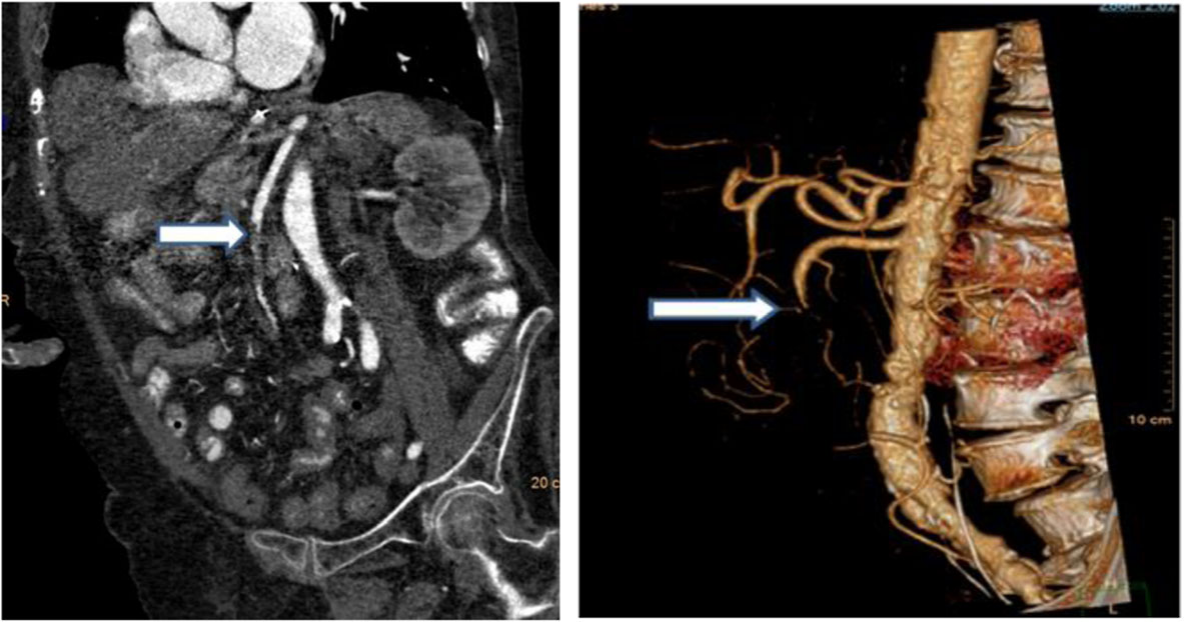
Selected image from a CTA scan of a patient with acute mesenteric ischemia secondary to occluded SMA from an embolic source (arrow). 3D reconstruction is demonstrates mid occlusion of SMA (arrow)

Delay in diagnosis is the dominant factor that accounts for continued mortality rates as high as 30–70% despite vast clinical experience and recognition of this entity [32, 33]. The multi-detector CTA has supplanted formal angiography as the diagnostic study of choice. Multi-detector computed tomography (MDCT) scanners are essential for the early diagnosis of AMI, but often require specialized personnel to perform and interpret the findings. 3D reconstruction is frequently helpful (Fig. 1). Volume rendering as in this image is now a semi-automatic workflow component of many CT machines. These can aide remote communities with less experienced staff.

In the presence of advanced AMI, the CTA findings reflect irreversible ischemia (intestinal dilatation and thickness, reduction or absence of visceral enhancement, pneumatosis intestinalis, and portal venous gas) and free intraperitoneal air [34].

Comprehensive biphasic CTA includes the following important steps:

- Pre-contrast scans to detect vascular calcification, hyper-attenuating intravascular thrombus and intramural hemorrhage.
- Arterial and venous phases to demonstrate thrombus in the mesenteric arteries and veins, abnormal enhancement of the bowel wall, and the presence of embolism or infarction of other organs.
- Multi-planar reconstructions (MPR) to assess the origin of the mesenteric arteries [35].

CTA should be performed despite the presence of renal failure, as the consequences of delayed diagnosis, missed diagnosis, or mismanagement are far more detrimental to the kidneys and the patient then exposure to the iodinated contrast agent. A recent study found that in 27 of 28 patients (96.4%) MDCT correctly diagnosed AMI (specificity of 97.9%) [16, 36]. A sensitivity of 93%, specificity of 100%, and positive and negative predictive values of 100 and 94%, respectively, were achieved [37, 38].

**Fig. 2:**
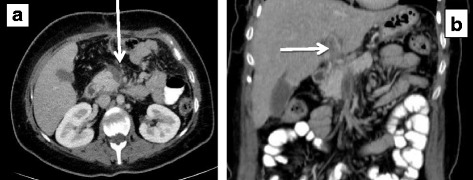
30-year-old patient with acute superior mesenteric vein **a** and portal vein thrombosis **b** due to hypercoagulable state. No signs of bowel ischemia were noted, and the patient was treated successfully with long-term anticoagulation

In NOMI CTA may demonstrate bowel ischemia and free fluid in the face of patent mesenteric vessels. In MVT, the most common positive radiological finding on venous phase CTA is thrombus in the superior mesenteric vein on venous phase CTA (Fig. 2). This has been described as the target sign [39].

Associated findings that suggest MVT include bowel wall thickening, pneumatosis, splenomegaly, and ascites [39]. Portal or mesenteric venous gas strongly suggests the presence of bowel infarction. Duplex ultrasonography has a limited role in this entity, but may be helpful if obtained early in chronic cases [22].

## Non-occlusive mesenteric ischemia (NOMI) should be suspected in critically ill patients with abdominal pain or distension requiring vasopressor support and evidence of multi-organ dysfunction. (Recommendation 1B)

Unexplained abdominal distension or gastrointestinal bleeding may be the only signs of acute intestinal ischemia in NOMI and may be undetectable in sedated patients in the ICU in approximately 25% of cases [40, 41]. Patients surviving cardiopulmonary resuscitation who develop bacteremia and diarrhea (with or without abdominal pain) should be suspected of having NOMI. Right-sided abdominal pain associated with the passage of maroon or bright red blood in the stool is highly suggestive of NOMI in these patients.

Gastrointestinal perfusion is often impaired early in situations of critical illness, major surgery or trauma, all of which are characterized by increased demands on the circulation to maintain tissue oxygen delivery [42]. This relative mesenteric hypoperfusion is often aggravated by an underlying hypovolemic or a low-flow state. In cases of intraabdominal hypertension, all of the structures within the abdominal cavity are compressed, and this will lead to regional hypoperfusion to the organs in the splanchnic bed. Such an effect is most pronounced in the liver due to its size. Animal studies have shown that even with intraabdominal pressure of only 10 mmHg, portal venous blood flow is reduced considerably, and that at 20 mmHg, the portal venous flow and hepatic arterial flow are reduced by 35 and 55%, respectively [43].

Most of the symptoms listed in this section are often not clinically apparent in a critically ill ventilated patient on ICU. Accordingly, any negative changes in patient’s physiology, including new onset of organ failure, increase in vasoactive support and nutrition intolerance should raise the suspicion of mesenteric ischemia.

## When the diagnosis of AMI is made, fluid resuscitation should commence immediately to enhance visceral perfusion. Electrolyte abnormalities should be corrected, and nasogastric decompression initiated. (Recommendation 1B)

Fluid resuscitation with crystalloid and blood products is essential for the management of the patient with suspected AMI. Preoperatively resuscitation is important to prevent cardiovascular collapse on induction of anesthesia. To guide effective resuscitation, early hemodynamic monitoring should be implemented [44]. Assessment of electrolyte levels and acid–base status should be performed. This is especially true in patients with AMI, where severe metabolic acidosis and hyperkalemia may be present due to underlying bowel infarction and reperfusion [45]. Vasopressors should be used with caution, and only to avoid fluid overload and abdominal compartment syndrome. Dobutamine, low dose dopamine, and milrinone to improve cardiac function have been shown to have less impact on mesenteric blood flow [46, 47]. The fluid volume requirement in these patients may be high, due to extensive capillary leakage, but extensive crystalloid overload should be avoided to optimize bowel perfusion [48]. The endpoints of therapy should address physiologic levels of oxygen delivery with continued monitoring of lactate level as an indication of improvement. Although in the past, supra-physiologic levels were advocated, current evidence does not support this concept [49].

## Broad-spectrum antibiotics should be administered immediately. Unless contraindicated, patients should be anticoagulated with intravenous unfractionacted heparin. (Recommendation 1B)

The high risk of infection among patients with AMI outweighs the risks of acquired antibiotic resistance, and therefore broad-spectrum antibiotics should be administered early in the course of treatment [50]. Intestinal ischemia leads to early loss of the mucosal barrier, which facilitates bacterial translocation and the risk of septic complications.

## Prompt laparotomy should be done for patients with overt peritonitis. (Recommendation 1A)

When physical findings suggestive of an acute intraabdominal catastrophe are present, bowel infarction already occurred, and the chance of survival in this patient population with significant associated comorbidity is dramatically reduced. There is overwhelming evidence in literature that peritonitis secondary to bowel necrosis mandates surgery without delay.

The goal of surgical intervention for AMI includes:

1. Re-establishment blood supply to the ischemic bowel.
2. Resection of all non-viable regions.
3. Preservation of all viable bowel.

Intestinal viability is the most important factor influencing outcome in patients with AMI. Non-viable intestine, if unrecognized, results in multi-system organ dysfunction and ultimately death. Prompt laparotomy allows for direct assessment of bowel viability.

After initial resuscitation, midline laparotomy should be performed followed by assessment of all areas of the intestine with decisions for resection of all clearly necrotic areas. In cases of uncertainty, intraoperative Doppler may be helpful, as the presence of Doppler signals over distal branches of SMA facilitates bowel conservation, avoiding long-term disability. The SMA is easily palpated by placing fingers behind the root of the mesentery. The SMA is identified as a firm tubular structure, which may or may not have a palpable pulse. Otherwise, the SMA can also be reached by following the middle colic artery where it enters the SMA at the mesentery. Direct sharp dissection, exposing the artery from its surrounding mesenteric tissue, is required for proper exposure to perform revascularization. In cases of diagnostic uncertainties, arteriogram is the study of choice. It can be done intraoperatively especially in hybrid suites.

**Fig. 3:**
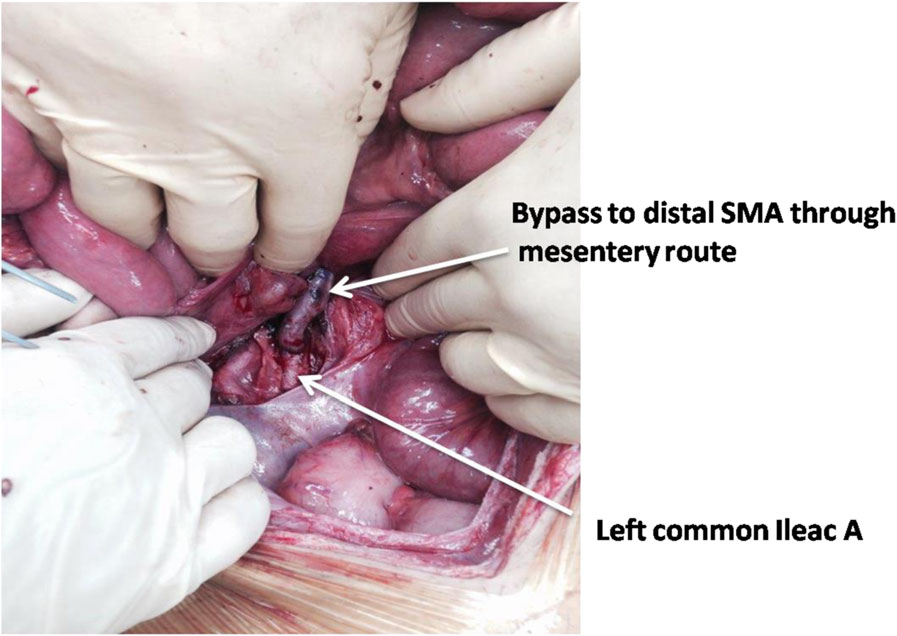
Patient with acute thrombosis of SMA underwent left ileo–SMA bypass with a common femoral vein graft

Different techniques of blood flow restoration are used depending on the pathophysiology of the AMI. Embolectomy and either primary or patch angioplasty is a well-established definitive treatment for SMA emboli. On the other hand, thrombosis of the SMA at the origin of aorta (a common pathology in diffuse aterosclerosis) will require a bypass procedure. However, it increases the magnitude of the procedure and may require prosthetics in the presence of contaminated field. One option is a retrograde bypass from the iliac artery to the distal SMA using the femoral vein or a synthetic graft (Fig. 3).

Neither NOMI nor MVT typically require vascular repair. Full dose anticoagulation should be initiated on all patients prior to the surgical procedure. Unfractioned heparin is effective and easy to manage, especially in patients with acute kidney failure.

## Endovascular revascularization procedures may have a role with partial arterial occlusion. (Recommendation 1C)

Several case series using endovascular techniques in combination with pharmacologic therapy have been reported recently. It should be emphasized, however, that any evidence of bowel ischemia or infarction precludes the use of thrombolytic therapy. At this time, these techniques have been attempted in very early, cases of AMI, and the role of such procedures remains to be determined [51, 52]. Other contraindications to thrombolytic therapy include recent surgery, trauma, cerebrovascular or gastrointestinal bleeding, and uncontrolled hypertension [53].

In recent retrospective series of 679 patients with AMI and vascular intervention (both open and endovascular) endovascular treatment performed in 24% (165 patients). The technique was successful in 87% of the patients, and in-hospital mortality was lower than among those who underwent open procedure (25 vs. 40%) [10]. Again, this report emphasized that only patients who did not require open emergent intervention are suitable for this technical approach to revascularization.

Endovascular embolectomy may be achieved by percutaneous mechanical aspiration or thrombolysis and permits percutaneous transluminal angioplasty, with or without stenting in case series of patients with CTA evidence of acute partial or complete occlusion of the SMA (either the main trunk or branch) and without no clinical or imaging evidence of advanced bowel ischemia. Complete technical success was achieved in 28% of cases; all of these had occlusion of the main SMA trunk [54–57].

There are no randomized controlled trials comparing laparotomy versus endovascular treatment as a first line strategy for the management AMI [10, 58, 59]. The most important argument in favor of the early laparotomy approach is the ability to assess bowel viability directly and thereby, minimizing delays in restoring mesenteric blood flow. In one retrospective series, the authors documented that 1/3 of patients managed with endovascular therapy avoided laparotomy [10]. In cases of endovascular approach, the use of laparoscopy to assess bowel function may be a reasonable addition [60].

Centers of excellence equipped with hybrid operating rooms may provide further data supporting the use of an endovascular strategy [61].

## Damage control surgery (DCS) is an important adjunct for patients who require intestinal resection due to the necessity to reassess bowel viability and in patients with refractory sepsis. Planned re-laparotomy is an essential part of AMI management. (Recommendation 1B)

Damage control laparotomy strategy (abbreviated laparotomy) was accepted for trauma over 30 years ago and was found to be an important option in the patient with AMI. Damage control is the surgical modality of choice in the critically ill patient with AMI for physiological and technical reasons. The decision to implement the DCS mode should be made early based upon the response to resuscitation and ongoing physiology, as this has been associated with improved mortality [62]. Advanced age is not a contraindication to DCS as good outcomes have been observed in the elderly [63].

Planned second look techniques are required after restoration of SMA flow, with or without resection of ischemic bowel (and no anastomosis or stoma) following resuscitation in intensive care unit [64, 65]. Given frequent uncertainty with regard to bowel viability, the stapled off bowel ends should be left in discontinuity and re-inspected after a period of continued ICU resuscitation to restore physiological balance. Often, bowel which is borderline ischemic at the initial exploration will improve after restoration of blood supply and physiologic stabilization. Of note, however, multiple adjuncts have been suggested to assess intestinal viability, but none have proven to be uniformly reliable [66, 67].

Most often, re-exploration should be accomplished within 48 h and decisions regarding anastomosis, stoma, or additional resection can be made with plans for sequential abdominal closure.

In a review of 43 patients undergoing open mesenteric revascularization, the authors noted that 11 of the 23 patients undergoing a second-look operation required bowel resection [20]. The bowel in these patients is often very swollen and at high risk for anastomotic leak. Recent studies suggest that careful hand sewn techniques are preferable to the use of staples in this group [68, 69].

These patients often suffer from acidosis, hypothermia, and coagulation abnormalities, which require prompt and ongoing correction. Physiologic restoration is multi-factorial and includes careful and limited crystalloid infusion to avoid abdominal compartment syndrome, frequent monitoring of lactate clearance and central venous oxygen saturation as an indication of satisfactory cardiac output, and the use of viscoelastic techniques (TEG, ROTEM) to assess coagulation status and guide ongoing blood product administration. Recent evidence suggests that peritoneal resuscitation techniques may aid in this process [70, 71].

Various techniques of open abdomen have been described. The author’s preferred mechanism is a simple plastic drape over the bowel, covered with a sterile towel and the use of Ioban over the abdomen. After the initial laparotomy, abdominal closure via negative pressure wound therapy is most commonly used. The open abdomen may help reduce the risk of abdominal compartment syndrome in patients requiring prolonged resuscitation. Various abdominal closure techniques have been described, however, the guiding principle is constant traction on the fascia to facilitate closure [72–75].

## Mesenteric venous thrombosis can often be successfully treated with a continuous infusion of unfractionated heparin. (Recommendation 1B)

MVT has a distinctive clinical finding on CTA scan, and when noted in a patient without findings of peritonitis, non-operative management should be considered. The first-line treatment for mesenteric venous thrombosis is anticoagulation. Systemic thrombolytic therapy is rarely indicated. When clinical signs demand operative intervention, one should resect only obvious necrotic bowel and employee damage control techniques liberally, since anticoagulation therapy may improve the clinical picture over the ensuing 24–48 h. Early use of heparin has been associated with improved survival [76].

Patients with peritonitis require emergency surgery. Intraoperative management is dictated by the surgical findings, which range from a segmental infarction of small bowel to necrosis of the entire bowel, with or without perforation. The aim of resection is to conserve as much bowel as possible. Second-look laparotomy, 24–48 h later, may avoid the resection of bowel that may be viable. A second-look procedure is mandatory in patients who have extensive bowel involvement.

Most published data on interventional radiological treatments for MVT are from small case series. Systemic intravenous tPA has been successfully reported [77]. Trans-jugular intrahepatic portosystemic shunt can be used for MVT with the rationale of decreasing portal pressure, which works as a vacuum of clot fragments and improves the effectiveness of thrombolysis in the case of acute thrombosis [78–80].

Supportive measures include nasogastric suction, fluid resuscitation, and bowel rest.

## When NOMI is suspected, the focus is to correct the underlying cause wherever possible and to improve mesenteric perfusion. Infarcted bowel should be resected promptly. (Recommendation 1B)

Management of NOMI is based on treatment of the underlying precipitating cause. Fluid resuscitation, optimization of cardiac output, and elimination of vasopressors remain important primary measures. Additional treatment may include systemic anticoagulation and the use of catheter-directed infusion of vasodilatory and antispasmodic agents, most commonly papaverine hydrochloride [81]. The decision to intervene surgically is based on the presence of peritonitis, perforation, or overall worsening of the patient’s condition [47].

If a patient presents with peritoneal signs, an exploratory laparotomy is required for resection of frankly necrotic bowel. Unfortunately, these patients are often in critical condition and the mortality remains very high (50–85%) [9]. Damage control mode is an important adjunct, given the critical state of these patients.

## The finding of massive gut necrosis requires careful assessment of the patients underlying co-morbidities and advanced directives in order to judge whether comfort carries the best treatment. (Recommendation 1C)

In cases of extensive infarction of most of the small bowel with or without a portion of the colon, the surgeon could face with a philosophical decision whether to do anything. Resection of the entire involved bowel will result in short bowel syndrome with its serious associated consequences. This may not be a preferable state, particularly in elderly infirm patients, who may not tolerate long-term parenteral nutrition. A preoperative discussion with the patient and the patient’s family concerning these issues is warranted and often necessary peri-operatively as well so that an agreeable plan can be reached [82].

## Conclusions

AMI is a true surgical emergency. First and foremost, important evidence is a high index of suspicion based on the combination of history of abrupt onset of abdominal pain, acidosis, and organ failure. This clinical scenario should prompt imaging (CTA) in order to establish the diagnosis. In parallel with rapid resuscitation and after careful assessment of the CTA, the patient should be explored to assess bowel viability, re-establish vascular flow, and resect non-viable bowel. Subsequently, the employment of damage control techniques and continued critical care resuscitation is essential. Planned re-assessment of the bowel with further resection or anastomosis and stoma as needed is integral. Close cooperation between acute care surgeons, radiologists, anesthetists, and the vascular surgeons is essential.

## Appendix

Current recommendations:

### Statement 1

Severe abdominal pain out of proportion to physical examination findings should be assumed to be AMI until disproven. (Recommendation 1B)

### Statement 2

Clinical scenario differentiates AMI as mesenteric arterial emboli, mesenteric arterial thrombosis, NOMI, or mesenteric venous thrombosis. (Recommendation 1B)

### Statement 3

Conventional plain X-ray films have limited diagnostic value in evaluating AMI, although signs of intestinal perforation may be seen. (Recommendation 1B)

### Statement 4

There are no laboratory studies that are sufficiently accurate to identify the presence or absence of ischemic or necrotic bowel, although elevated l-lactate and D-dimer may assist. (Recommendation 1B)

### Statement 5

Computed tomography angiography (CTA) should be performed as soon as possible for any patient with suspicion for AMI. (Recommendation 1A)

### Statement 6

Non-occlusive mesenteric ischemia (NOMI) should be suspected in critically ill patients with abdominal pain or distension requiring vasopressor support and evidence of multi-organ dysfunction. (Recommendation 1B)

### Statement 7

When the diagnosis of AMI is made, fluid resuscitation should commence immediately to enhance visceral perfusion. Electrolyte abnormalities should be corrected, and nasogastric decompression initiated. (Recommendation 1B)

### Statement 8

Broad-spectrum antibiotics should be administered immediately. Unless contraindicated, patients should be anticoagulated with intravenous unfractionated heparin. (Recommendation 1B)

### Statement 9

Prompt laparotomy should be done for patients with overt peritonitis. (Recommendation 1A)

### Statement 10

Endovascular revascularization procedures may have a role with partial arterial occlusion. (Recommendation 1C)

### Statement 11

Damage control surgery is an important adjunct for patients who require intestinal resection due to the necessity to reassess bowel viability and in patients with refractory sepsis. Planned re-laparotomy is an essential part of AMI management. (Recommendation 1B)

### Statement 12

Mesenteric venous thrombosis can often be successfully treated with a continuous infusion of unfractionated heparin. (Recommendation 1B)

### Statement 13

When NOMI is suspected, the treatment focus should be to correct the underlying cause and to restore mesenteric perfusion. Infarcted bowel should be resected promptly. (Recommendation 1B)

### Statement 14

The finding of massive gut necrosis requires careful assessment of the patients underlying co-morbidities and advanced directives in order to judge whether comfort carries the best treatment. (Recommendation 1C)

## Abbreviations

alpha–GST: Serum alpha-glutathione S-transferase
AMI: Acute mesenteric ischemia
CABA: Cobalt-albumin binding assay
CMI: Chronic mesenteric ischemia
CTA: Computed tomography angiography
DCS: Damage control surgery
I-FABP: Intestinal fatty acid binding protein
MDCT: Multi-detector computed tomography
MPR: Multi-planar reconstructions
NOMI: Non-occlusive mesenteric ischemia
NPWT: MVT: Mesenteric venous thrombosis
SMA: Superior mesenteric artery
tPA: Tissue plasminogen activator
WSES: World Society of Emergency Surgery
